# Research on quantitative assessment of translation quality from the perspective of phraseology

**DOI:** 10.1371/journal.pone.0318804

**Published:** 2025-02-13

**Authors:** Bojia He, Jinquan Wang, Yizhe Wang

**Affiliations:** 1 College of International Studies, Yangzhou University, Yangzhou, Jiangsu, China; 2 Department of Computer Science, Rice University, Houston, Texas, United States of America; Faculty of Electrical Engineering, Computer Science and Information Technology Osijek, CROATIA

## Abstract

Supported by the relevant theories of phraseology, this study examined the translation quality of three genres of explanatory, argumentative, and narrative essays at the phrase level and aimed to construct a translation quality assessment model. In this study, a total of six variables were extracted from both linguistic form and linguistic meaning in strict accordance with the phrase screening criteria, among which the linguistic form features contained a 2-4 gram match degree and the linguistic meaning features contained a part-of-speech tagged 2-4 gram match degree. The results showed that, first, bigram-related variables were the strongest predictors of translation scores for the three genres. The trigram-related variables were slightly weaker, and the fourgram-related variables were the lowest. The bigram match degree had the highest correlation coefficient of .752** with explanatory text translation scores. Second, all translation quality assessment models of the three genres fit well, with the highest correlation coefficient for the explanatory text model, R = 0.820, R^2^ = 0.673, followed by argumentative text, and the lowest for narrative text. This study realized the automatic assessment of the meaning and form of translations of different genres, which had certain theoretical and practical significance.

## Introduction

Psycholinguistics research has found that phrases are the principal units of language storage and extraction. When people reflect and express themselves, they always use phrases as the carriers of meaning construction to form their ideas and export their words. Sinclair believed that meaning units were the embodiment of the co-option of grammar and lexical relations. Phraseological research based on corpus has found that “meaning units mostly appear in the form of phrases, and meaning is embodied in phrases formed by the co-option of multiple words and grammatical structures” [1]. From the viewpoint of phraseology, translation can be regarded as a product of the co-option of the original language meaning and the lexical and grammatical meanings of the translated language and their constituent meanings. Translation quality assessment is a core thesis in translation research, which has received extensive attention from translation theorists all over the world. Existing studies on translation quality assessment are dominated by qualitative assessment, and quantitative studies are rare. From the perspective of phraseology and by combing and analyzing phrases in Chinese students’ Chinese-to-English translations, this study explores the influence of relevant quantitative features based on Chinese students’ phrase use on the quantitative assessment of translation quality in translations of different genres. On the one hand, this study mines linguistic meaning and form variables related to the assessment of translation quality, expanding the method and scope of translation quality assessment. On the other hand, the relevant variables extracted in this study can realize the automatic assessment of linguistic meaning and form of translations at the phrase level, which has strong application value and practical innovation.

## Literature review

### Definition and scope of phrases

In traditional phraseology, top-down rational analyses were primarily used, with research focusing on proverbs, idioms and verb phrases. Russian scholars Vinogradov and Amosova classified phraseological units on the basis of the criterion of “fixedness, rigidity” and proposed the “phrase continuum” [2]. Vinogradov believed that the core of phraseology was idiomatic phraseological units and that free phrases that were semantically transparent and formally variable should be excluded. Vinogradov classified phraseological units into “phraseological combinations”, “phraseological unities” and “phraseological fusions” based on the relationship between the meaning of the whole part and that of its components. Among them, “phraseological combinations” were formed by combining a constituent with direct meaning and a constituent with figurative meaning. “Phraseological unities” were relatively more numerous in English, and the meaning of “phraseological unities” was usually expressed in terms of the metaphorical meaning of the whole phrase. “Phraseological fusions” represented the highest degree of fusion between different constituents, i.e., the meaning of the phrase as a whole had completely absorbed the meaning of the constituents. Usually, such phraseological units cannot be understood directly. In addition to the research system of Russian scholars, Cowie [3–8] divided word combinations into three categories, including “free combinations”, “compositions” and “formulae”. In the phraseological continuum put by Cowie, those word combinations that are associated freely are also excluded. However, idioms with a high degree of formality are only a small part of modern language use. In summary, the phraseology pioneered by Russian scholars established a set of semantic and structural criteria for phraseological research, thereby laying the foundation for subsequent research in this field. Some European and American scholars inherited the traditional paradigm of phraseology from Russian scholars. To some extent, traditional phraseological researchers have neglected the importance of “collocation”. They have concentrated mainly on restricted collocations, i.e., idioms and relatively fixed expressions. The present corpus reveals a reduced frequency of the occurrence of the phrases prescribed by traditional phraseology. Furthermore, traditional phraseology has paid little attention to the general collocations, which are more free and more collocatable.

Based on the traditional descriptive framework and theoretical system of phraseology, Sinclair put forward corpus-based big data phraseological research and summarized a new theoretical framework of phraseology, which consists of three levels, namely, contextual settings, phraseological items, and lexical items [9]. Phraseological items were collocational frameworks previously proposed by Sinclair, which defined the concept of “phrase”. Renouf [10] noted that the linguistic skeleton of a complete phrase was a discontinuous sequence of two noncontiguous “grammatical words”, which were also called “closed classes of small words”. This framework provided space for other open-class words to be optionally filled in. Sinclair [11] referred to this combination as a “phrase term”. At the present stage, corpus phraseological researches focus not only on the study of language ontology, such as “formulated language” [12] and “Construction Grammar” [13], but also on the study of language use, such as cross-language comparison and cross-cultural communication [14–16].

Granger and Paquot [17] conducted a study on the content categorization of phraseological units and proposed criteria for the classification of 16 subcategories into three broad categories. Based on this, Wei [18] classified the various types of phraseological units in phraseological research into 11 categories from four levels: phrase, clause, sentence and stem, by synthesizing the “phrase continuum” in traditional phraseology and the “phraseological items” in corpus phraseology. Specific classifications and examples are shown in Table 1.

**Table 1:** Phrase categories and subcategories.

Phrase subcategories		Samples
Word Collations	N. N.	career development, team spirit, post box
	Adj. N.	little dog, red house, beautiful view
	V. N.	make a decision, play basketball, read books
	V. Adv.	apologize profusely, listen carefully, recommend strongly
	Int. Adj.	fully appreciate, significantly different, awfully sorry
Fixed-order Words	Irreversible Binomials	men and women, rise and fall, home and abroad
	Irreversible Trinomials	centre, up and down; hook, line and sinker
Stereotyped Similes	As Adj. As N.	as white as snow, as black as night, strong as an ox (The first “as” in this structure can be omitted.)
	V. Like N.	cry like a baby, sleep like a log, work like a dog
Phrasal Verbs	V. Prep.	account for, look up, take in
	V. Adv.	bring about, look around, look after
	V. Adv. Prep.	come up with, add up to, take care of
	V. Adj.	go wrong, turn left, go straight
	Others	get in touch with, look forward to, keep an eye out for
Complex Prepositions	Prep. N. Prep.	in terms of, in view of, on account of
	Prep. Adj./Adv. Prep.	as opposed to, as far as, as well as
	Adj. Prep.	regardless of, prior to, devoid of
	Adv. Prop.	apart from, rather than, along with
	Prep. Prep.	but for, except for, as to
Complex Conjunctions		as soon as, as long as, given that
Linking Adverbials	Enumeration and Addition	for one thing, for another, in addition
	Conclusion	to sum up, in brief, to conclude
	Outcome/Inference	that is, that is to say, in other words
	Consequence	as a result, consequently, to this end
	Contrast/Concession	by contrast, on the other hand, on the contrary
	Transition	meanwhile, at the same time, by the way
Textual Sentence Stems		We will argue that..., It will be shown that..., The paper discusses....
Formulae		good morning, nice to meet you, I am sorry
Commonplaces		It matters little, It is nothing to me, There is no alternative
Idiom		keep the lid on, the cutting edge, hold your horses

### Review on translation quality assessment

With the gradual cross-fertilisation between translation and other disciplines, the boundaries of translation, both within and outside the field of translation, are gradually becoming blurred and the topics of translation research are constantly enriched. Translation quality assessment is one of the most controversial issues that has been widely and persistently studied by scholars [19–27] However, to some extent, translation quality assessment often lacks objective analysis and is mostly based on intuition and impression [28]. Therefore, quantitative translation quality assessment has become a research trend in recent years. Quantitative assessment of translation quality refers to the use of quantitative methods to turn abstract qualitative judging criteria into concrete and operable quantitative models. The most important starting point of quantitative assessment is the pursuit of objectivity, i.e., to strive for objective methodology and scientific theory.

In terms of theoretical underpinnings, theoretical support for translation quality assessment has moved from a single theory of translation to the integration of linguistics and translation. With the shift of assessment theory to linguistics, three influential approaches to translation quality assessment have emerged, namely Functional-Pragmatics Principle(Julian House), Discourse Type Principle (Christina Reiss) and Argument Schema Theory (Malcolm Williams). These three classic approaches to translation quality assessment avoided the subjectivity of traditional intuition to a certain extent and gradually approached scientific and objective assessment. However, their focus was still on qualitative analysis and quantification was insufficient. Subsequently, the Canadian Language Quality Measurement System (SICAL), the Localisation Industry Standards Association (LISA QA Model), SAE J2450, Multidimensional Quality Metrics (MQM) and other translation quality assessment methods emerged, which were based on error analysis. Of these, MQM was developed by Forschungszentrum für Künstliche Intelligenz (DFKI) after integrating LISA, SAE J2450 and other methods. The model is comprehensive, versatile and customisable [29]. Overall, the error analysis-based assessment model shows a tendency towards quantitative evaluation, but is still somewhat subjective in terms of assigning specific weights.

From a methodological perspective, translation quality assessment has moved from intuitive judgement to mathematical and computational science. Traditional translation quality assessment is intuitive assessment, that is, the quality of the translation depends on the personal experience and subjective judgement of the assessor, which is what House [30] refers to the “mentalist views of translation quality assessment”. In order to avoid the subjectivity of assessment, some scholars integrated fuzzy mathematical theory with translation quality assessment, quantitatively evaluated the quality of translations in terms of syntactic structure, lexical collocation, style hierarchy, stylistic hierarchy, and constructed mathematical models. Then, they creatively put forward a quantitative assessment model that was easily programmed by computers [31–34]. In recent years, research on the assessment of translation quality based on fuzzy mathematical theory has not yielded any significant breakthroughs [35]. The rapid development of computer technology, large language model and artificial intelligence technology has provided new technical support for methods of translation quality assessment. A considerable number of scholars have conducted research on translation quality assessment using a variety of methods, including natural language processing, computational linguistics, corpus linguistics, deep learning, and neural network. Wang [36] assessed the quality of translation from both semantic and formal aspects. He extracted a total of 29 significantly relevant text features and constructed an automatic scoring model for Chinese-to-English translation. Subsequently, Wang et al. further mined relevant textual features from the perspectives of vocabulary, phrases, syntax and chapters to improve the automatic scoring model for translation quality [37–40]. De Sutter et al. [41] attempted to statistically analyse linguistic features such as the type-token ratio, lexical density, word frequency and other linguistic features with a view to judge the quality of the translation. The continuous application of deep learning technology has led to the emergence of numerous deep neural network-based translation quality assessment methods, including QUETCH (quality estimation from scratch) [42], BERT (bidirectional encoder representation from transformers) [43], TransQuest [44], and others.

Furthermore, the automated assessment of machine translation serves as a valuable reference point in translation quality assessment. among the existing machine translation assessment systems, the assessment method and standards of BLEU (bilingual evaluation understudy) and the NIST (national institute of standards and technology) were the most representative. Both the above assessment methods were inspired by the approach of N-gram matching. The N-gram matching can be considered an approach that a machine matches the translation to be tested with the reference translation at the N-gram (1-4 grams) level. If the same N-gram appears during the matching process, it defaults to the same phraseological unit and assigns a value of 1, otherwise it assigns a value of 0. This method has been demonstrated to be effective in evaluating the similarity between the translation to be tested and the reference translation in related studies [45]. Nevertheless, it is important to note that the similarity of linguistic items in the natural language should be a continuously changing value that infinitely tends to the extremes of 0 and 1. It would be counterintuitive to judge the similarity by two extreme values. Furthermore, the method merely extracts the N-grams in the translation without implementing a filtration process. The extracted phrases contain invalid phrases composed of function words, which cannot reflect semantic similarity at a deep level.

Consequently, from the perspective of phraseology, this study adopted the N-gram approach as a prototype, utilizing a multifaceted approach that encompassed the filtering of N-grams and the part-of-speech tagging of N-grams. It further delved into the correlation between phraseological units and translation quality from the points of linguistic meaning and linguistic form, striving to construct an automated scoring model for translation quality.

### Research design

This study took phrases in Chinese EFL learners’ Chinese-English translations as the object of study. From the perspective of lexical collocation and grammatical structure, this study examined the influence of phraseological units on the quantitative assessment of translation quality by integrating vocabulary, grammar and meaning.

### Research questions

This study addressed the following two questions:

Question 1: What is the correlation between translation quality assessment variables based on phraseological measuring features and translation quality?
Question 2: What is the predictive power of translation quality assessment variables based on phraseological measuring features in scoring models?

### Research corpus

The translation corpus collected for this study was from the *Parallel Corpus of Chinese EFL Learners* (PACCEL), which contained three genres of narrative, expository, and argumentative essays. The translations in this study included 300 narrative essays, 336 expository essays, and 257 argumentative essays, all of which were Chinese-English translated texts with a time limit for translation of 60 minutes. Chinese large-scale tests that contain translation tests include College English Test Band4 and Band6, Test for English Majors-Band 8, The National Entrance Examination for Postgraduate, China Accreditation Test for Translators and Interpreters (CATTI). A synthesis of the aforementioned tests indicates that translation tasks in the large-scale tests can be primarily classified into two categories: literary and non-literary genres. PACCEL contains a comprehensive array of English-Chinese/Chinese-English interpreting and translating materials on a vast range of subjects. The students’ translations of non-news narrative essays can be classified as literary, whereas those of argumentative and expository essays are non-literary. In order to guarantee the feasibility of the study and to meet the requirement for large-scale translation tests, all three genres were selected as the corpus for this study.

### Research tools

The tools used in this study mainly contain text preprocessing tools, text feature extraction tools and data analysis tools. The text preprocessing tools include part-of-speech tagging software (Claws7), as well as self-written tools for text cleaning and word shape reduction. The text feature extraction tool includes Antconc 4.2.0. The data analysis tool is SPSS 27.0.1.

### Phrase extraction and filtering

In this study, the N-grams were extracted from the translations to be tested and the best translations in each of the three genres by using Antconc 4.2.0, with the frequency set to 2. Due to the difficulty of finding matches for the fivegram in the translations to be evaluated [46], “N” was set to 2-4. Synthesizing the views on phrase definition and filtering from scholars such as Coulmas [47], Cock [48], Biber and Barbieri [49], Wei [50], Li and Zhao [51], and Li and Deng [52], combined with the actual situation of this study, this study defined the criteria for phrase filtering and elimination as the following three points. First, phrases consisting entirely of closed words, such as *with a*, *to the*. Second, phrases with breaks or small sentence fragments such as *level in s and*, *home is that it*. Third, phrases with names of people and places such as *nanjing chemistry*. The first two types of phrases have incomplete expressions of meaning and inchoate grammatical information. The third type of phrases are proper nouns, which have little relevance to the quantitative assessment of translation quality. If the above phrases are included in the scope of similarity examination, it may result in a decline in the correlation coefficient between the similarity outcomes and the human assessment. This phenomenon is analogous to the traditional N-gram method, and it somewhat reduces the effectiveness of phrases in judging the quality of translations.

By matching the screened 2-4 phraseological units and calculating the matching degree, the relationship between phrase content and translation quality can be examined at the semantic level. In addition, to investigate the relationship between phrase form and translation quality, this study utilized Claws4 part-of-speech tagging software to assign the part-of-speech of the translations to be tested and the best translations. Then, this study extracted the 2-4 phraseological units and calculated the degree of match, while there was no requirement to screen the phraseological units after part-of-speech tagging.

Based on the above criteria, a total of six text variables related to translation quality were extracted in this study, including the match degree of 2-4 phraseological units and the match degree of 2-4 part-of-speech tagged phraseological units, as shown in the following Table 2.

**Table 2:** Variables related to translation quality.

Variables on translation form	Variables on translation meaning
Match degree of bigram	Match degree of part-of-speech tagged bigram
Match degree of trigram	Match degree of part-of-speech tagged trigram
Match degree of fourgram	Match degree of part-of-speech tagged fourgram

## Results and discussion

### Correlation between the related measuring features of phrase meaning and translation scores

In this section, the quality of translation was measured at the semantic level by matching the 2-4 grams of the best translations and translations to be tested. Both translations were not part-of-speech tagged. The three translation quality assessment variables in this section are the match degree of 2-4 phraseological units. First, according to the definition of “phrase” in phraseology, the 2-4 phraseological units of the best translations were strictly screened. No matter how many times a phrase was repeated, it was only counted once. This approach was designed to prevent students from confusing the machine by copying phrases. Second, the number of phraseological units’ repetitions of each translation to be tested was counted to calculate the match degree of the 2-4 phraseological units of the translations to be tested and the best translations. Then, the relevant data were imported into SPSS to calculate the correlation between the degree of match and the scores of the translations to be tested. The definite data are as follows.

From the data in Table 3, it can be observed that the match degree of 2-4 phraseological units showed a significant positive correlation with the translation scores of all three genres. In terms of phrase length, the correlation between the bigram match degree and the translation grades of the three genres is relatively prominent, followed by the trigram match degree. The overall correlation between the fourgram match degree and the translation grades is the lowest. Among the bigram match degrees, the correlation with the expository translation grades is the highest, .752**, which is the highest among all the correlation coefficients for the three genres. In addition, the correlation with the narrative translations is the lowest at .279**. In terms of the trigram match degree, the highest correlation coefficient is for argumentative translations, .703**. The lowest is for narrative texts, .156**, which is the lowest correlation coefficient value of all three genres. The correlation coefficients with the three genres, in descending order of match degree, were .554** for argumentative essays, .473** for expository essays, and.163** for narrative essays. With regard to different genres, the mean values of the correlation coefficients of the degree of matching between explanatory, narrative, and argumentative texts and 2-4 phraseological units are 0.617, 0.199, and 0.630, respectively. The correlation between the results of translations of expository and argumentative essays and the match degree of 2-4 phraseological units are both relatively high, far exceeding the correlation between the results of translations of narrative essays and the match degree of phrases of different lengths. In the narrative essays, the correlation between the narrative translation scores and the match degree of bigrams is the highest, at only .279**, which is less than the lowest correlation coefficient between the explanatory and narrative essays.

**Table 3:** Correlation between the match degree of 2–4 phraseological units and translation scores.

Genre	Bigram match degree	Trigram match degree	Fourgram match degree
Explanatory Essay	0.752**	0.626**	0.473**
Narrative Essay	0.279**	0.156**	0.163**
Argumentative Essay	0.633**	0.703**	0.554**

** Indicates that the correlation coefficient is significant at the 0.01 level (2-tailed).

Overall, the correlation between the three translation quality assessment variables in this section and the three genres of translation scores is as follows: bigram match degree, trigram match degree and fourgram match degree from high to low, respectively. There are two reasons to explain the above results. First, the number of bigrams in the three genres slightly exceeds that of trigrams and fourgrams. The number of phraseological units that can be extracted from the translated texts is negatively correlated with the length of the phrases. That is to say, the longer the length is, the fewer the number of phrases extracted. Second, bigrams are marginally less impacted by synonyms in comparison to trigrams and fourgrams. The N-gram match approach utilized in this study merely counts exact repetitions of phrases, which cannot take into account the influence of synonyms on the phrases. Furthermore, bigrams have been demonstrated to offer superior capabilities in measuring the fluency of translations. As the size of N-grams increases, the accuracy of N-grams for the evaluation of translations experiences an exponential decrease. The findings presented in Table 3 are consistent with the data reported in Papineni et al.’s study in 2002. Based on this, bigrams perform better than trigrams and fourgrams in measuring the quality of translation. For example:

ST1:好好学习, 遵守纪律
TT1: work hard and be disciplined
TT2: study hard and observe discipline

The transliteration process is be somewhat influenced by the original text, which is less creative than writing. However, the use of synonyms ensures a greater variety of forms in the translated texts. As can be seen from ST1, the same text can produce two translation results. According to the phrase extraction rules of this study, two pairs of bigrams, “work hard” “study hard” and “be disciplined” and “observe discipline”, are affected by only one synonym. However, if the bigrams are expanded into trigrams and fourgrams, the potential for combination of phraseological units increase with them. This augmentation is accompanied by a significant escalation in the influence of these units exerted by synonyms.

Additionally, among the three genres, the correlation between the scores of translations and the match degree of 2-4 phraseological units is lower in narrative essays than in explanatory and argumentative essays. The original and translated languages of narrative texts were rich in content and diverse in expression. However, the language of expository and argumentative texts is plain and concise. Their forms of expression are not as complex as those of narrative texts. Therefore, the probability of synonyms or synonymous expressions in the translation of narrative essays is higher than that of explanatory texts and argumentative texts.

A thorough analysis of the original texts of the three genres reveals that the average sentence length of explanatory essays is the highest at 50.33, followed by narrative essays at 38.44, and argumentative essays at 22.73. Explanatory essays are characterized by a high degree of syntactic complexity, exhibiting a combination of simple and compound sentences, short and long sentences, and a high prevalence of dependent constituents in subordinate clauses. This linguistic complexity facilitates the identification of specific syntactic patterns. As illustrated in ST1, the sentence is more protracted, and the dependent components include coordinative constituents, appositives and adverbials of purpose. In contrast, argumentative essays are distinguished by their use of refined language, robust reasoning, and a high degree of condensation, resulting in an average sentence length that is the shortest of the three types of essay. In contrast, narrative essays are characterized by the use of vivid language and a greater prevalence of modification. The average sentence length of narrative essays exceeds that of argumentative essays. A comparison of ST2 and ST3 with ST4 reveals that, although argumentative essays are concise, they predominantly utilize four-word phrases and exhibit intricate syntactic structures. Although, there are abundant vocabulary and long sentences in narrative essays, coordinate components account for more, which reveals that the syntactic complexity of narrative essays is slightly less than that of argumentative essays.

ST1: 为了迎合国际上消费品“回归大自然”的发展趋势, 该厂开发研制了具有当代最新科技水平, 全国牙膏行业唯一发明专利产品——丝素牙膏, 引进国内外客户的极大兴趣和关注。(Explanatory Essay)
ST2: 名人必须细心思考问题, 谨言慎行。(Argumentative Essay)
ST3: 名人一旦说话出错, 便成为笑料。(Argumentative Essay)
ST4: 父母的回信上, 除了老生常谈的好好学习, 遵守纪律外, 还有一些令大学教授都为之皱眉的离奇的事。(Narrative Essay)

Influenced by the limitations of the N-gram match approach, the repetition rate of 2-4 phraseological units in narrative texts is lower than that of explanatory texts and argumentative texts, and the match degree of 2-4 phraseological units is a slightly weaker differentiator of narrative text translation quality. In all, the performance of the 2-4 phraseological unit match degree in narrative essays is not as good as that in explanatory and argumentative essays.

### Correlation between the related measuring features of the phrase form and translation Scores

In this study, the part-of-speech tagging software (Claws7) was utilized to lexically annotate the translations. This section examined the quality of translation at the language form level by matching the 2-4 phraseological units of both the part-of-speech tagged translation to be tested and the best translation. The three translation quality assessment variables in this section are the match degree 2-4 part-of-speech tagged phraseological units. Since the same part-of-speech tagged phrases can correspond to distinctive specific expressions, this section did not need to filter the phrases, and the number of phrase matches can be counted repeatedly. In addition, the calculation method of match degree and correlation in this section was consistent with the above section on phrase meaning measurement. The specific details are shown in Table 4.

**Table 4:** Correlation between the match degree of 2–4 part-of-speech tagged phraseological units and translation scores.

Genre	Part-of-speech tagged bigram match degree	Part-of-speech tagged trigram match degree	Part-of-speech tagged fourgram match degree
Explanatory Essay	0.684**	0.603**	0.456**
Narrative Essay	0.322**	0.219**	0.105
Argumentative Essay	0.435**	0.341**	0.388**

** Indicates that the correlation coefficient is significant at the 0.01 level (2-tailed).

As shown in Table 4, most of the match degrees of the 2-4 part-of-speech tagged phraseological units are significantly positively correlated with the translation grades of the three genres. Compared with the previous section, the correlation is slightly weaker than that between the matches of the 2-4 phraseological units and the translation grades of the three genres. In explanatory essays, the correlation between the match degree of part-of-speech tagged phraseological units and translation performance is more momentous, and the correlation coefficient is higher than that of narrative essays and argumentative essays as a whole. The part-of-speech tagged bigram has the highest match degree of .684**, and the part-of-speech tagged fourgram has the lowest match degree of .456**. The correlation between the match degree of 2-4 part-of-speech tagged phraseological units and the grades of argumentative translations is slightly weaker than that of explanatory essays. In descending order, it is the match degree of the part-of-speech tagged bigram, the match degree of the part-of-speech tagged trigram, and the match degree of the part-of-speech tagged fourgram. The correlation between the match degree of 2-4 part-of-speech tagged phraseological units and the scores of narrative translations is the weakest among the three genres. The highest is the match degree of the part-of-speech tagged bigram, only.322**, and the part-of-speech tagged fourgram match degree does not correlate with the grades of narrative translations.

Overall, in terms of different genres, the match degree of 2-4 part-of-speech tagged phraseological units has the strongest correlation with the translation scores of explanatory texts, followed by argumentative texts, and the lowest for narrative texts. In terms of phrase length, the correlation between the match degree of the 2-4 part-of-speech tagged phraseological units and the translation scores of the three genres is part-of-speech tagged bigram, part-of-speech tagged trigram, and part-of-speech tagged fourgram in descending order of strength. The above two points coincide with the conclusion of the phrase meaning part. The number of 2-4 part-of-speech tagged phraseological units extracted from the to-be-tested translations of three genres are, respectively, 300, 639, and 916 for explanatory essays, 402, 782, and 979 for narrative essays, and 280, 624, and 839 for argumentative essays. From the statistical data, it can be seen that the longer the length of the part-of-speech tagged phraseological units, the lower the repetition rate of their grammatical forms, the richer the grammatical expressions, and the higher the corresponding number that can be extracted. Therefore, the number of part-of-speech tagged fourgrams is the highest, followed by part-of-speech tagged trigrams and part-of-speech tagged bigrams. Meanwhile, the overall number of 2-4 part-of-speech tagged phraseological units is much higher than the number of 2-4 phraseological units because the 2-4 phraseological units after part-of-speech tagging have not been screened. Despite the fact that the number of 2-4 part-of-speech tagged phraseological units is large, the same grammatical collocation can have a variety of phrase expressions. For example, the CLAWS7 produced the part-of-speech tagged bigram “AT NN”, whose concrete expressions in the translations can be “every coin” or “every sword”; the part-of-speech tagged trigram “APPGE NN T”, whose concrete expressions in the translation can be “your pen to” or “our pen to”. Thus, only considering the match degree of the grammatical form of the phraseological units does not accurately examine the quality of translation. In summary, the correlation coefficient between the match degree of the 2-4 part-of-speech tagged phraseological units and the scores of the translations of the three genres is marginally lower than that of the match degree of the 2-4 phraseological units as a whole.

### Construction of the translation quality assessment model based on phraseological measurement features

In this section, a total of six translation quality assessment variables, which contained the match degree of the 2-4 phraseological units and the 2-4 part-of-speech tagged phraseological units, were comprehensively included in the translation quality assessment model. Then, stepwise regression analyses were carried out by using the data analysis software SPSS to further explore the contribution degree of the relevant phrase-based measurement features to the assessment of the translation quality of the three genres. Finally, the translation quality assessment model based on phraseological measurement features could be constructed.

As seen from the data in Table 5, the explanatory and argumentative essay models are better than the narrative essay model, and the correlation coefficients (R) for both explanatory and argumentative essays exceed 0.7, which is a good regression. Among them, the explanatory text model has the highest fit, R = 0.820, R^2^ = 0.673. The argumentative essay model is slightly weaker than the explanatory text, R = 0.757, R^2^ = 0.573. The narrative text has the weakest effect, with the lowest correlation coefficient, R = 0.343, R^2^ = 0.117. The performance of the models evaluating the translation quality for the three genres is in accord with the correlation of the relevant variables with the translation scores as described above. The explanatory text model performs the best, followed by the argumentative text model, and the narrative text model is the weakest.

**Table 5:** Translation quality assessment model of the three genres based on phraseological measurement features.

Genre	R	R Square	Adjusted R Square	Std Error of the Estimate
Explanatory Essay	0.820	0.673	0.669	6.950745
Narrative Essay	0.343	0.117	0.111	8.747639
Argumentative Essay	0.757	0.573	0.564	5.143282

The data related to the stepwise regression model of the explanatory essay are represented in Table 6. This model holds the highest degree of fit, with a total of four variables entering the regression equation, namely, the match degree of the bigrams and trigrams and the match degree of the part-of-speech tagged bigrams and part-of-speech tagged trigrams. Regardless of the fact that the positive and negative correlations of the variables showed different performances, all the ß values of the variables were in the same direction as the correlation coefficients. Meanwhile, the T test results are statistically significant. The model can explain 67.3% of the explanatory translation scores.

**Table 6:** Stepwise regression model of explanatory essay based on phraseological measurement features.

Model	Unstandardized Coefficients		Standardized Coefficients	T	Sig	VIF
	β	Std. Error	Beta			
(Constant)	22.167	1.764		12.568	0.000	
Bigram	153.761	22.132	0.424	6.948	0.000	3.552
Part-of-speech tagged bigram	140.739	18.095	0.549	7.778	0.000	4.759
Trigram	66.937	23.751	0.154	2.818	0.005	2.863
Part-of-speech tagged trigram	−199.196	71.119	−0.197	−2.801	0.005	4.696
Bigram = Match degree of bigrams	Part-of-speech tagged bigram = Match degree of part-of-speech tagged bigrams					
Trigram = Match degree of trigrams	Part-of-speech tagged trigram = Match degree of part-of-speech tagged trigrams					

By analysing the data in Table 7, it can be seen that among the three genres, the regression effect of the narrative essay model is slightly weak. There are only two relevant variables for the regression equation, which are the match degree of bigrams and part-of-speech tagged bigrams. The VIF values of all variables are less than 10, and the multicollinearity relationship between the two variables is weak, which complies with the requirements of this study. The model can explain only 11.7% of the narrative translation scores, which is the lowest explanation among the three genres.

**Table 7:** Stepwise regression model of narrative essay based on phraseological measurement features.

Model	Unstandardized Coefficients		Standardized Coefficients	T	Sig	VIF
	β	Std. Error	Beta			
(Constant)	50.407	2.903		17.363	0.000	
Bigram	40.537	19.459	0.143	2.083	0.038	1.471
Part-of-speech tagged bigram	77.418	21.999	0.241	3.519	0.001	1.471
Bigram = Match degree of bigrams			Part-of-speech tagged bigram = Match degree of part-of-speech tagged bigrams			

Table 8 shows the data related to the stepwise regression model for argumentative essay. Among the three genres, the number of variables entering the regression equation for argumentative essay is the largest, totalling five, which are the match degree of the bigrams and trigrams, the match degree of the part-of-speech tagged bigrams, part-of-speech tagged trigrams and part-of-speech tagged fourgrams. The model is consistent, only slightly weaker than the model for explanatory essay and better than the model for narrative essay. The regression effect of this model is significant, which can explain 57.3% of argumentative translation scores.

**Table 8:** Stepwise regression model of argumentative essay based on phraseological measurement features.

Model	Unstandardized Coefficients		Standardized Coefficients	T	Sig	VIF
	β	Std. Error	Beta			
(Constant)	31.904	2.018		15.807	0.000	
Bigram	46.611	16.128	0.194	2.890	0.004	2.433
Part-of-speech tagged bigram	88.684	17.226	0.552	5.148	0.000	5.563
Trigram	25.063	3.984	0.449	6.291	0.000	2.755
Part-of-speech tagged trigram	−418.833	94.078	−0.641	−4.452	0.000	11.206
Part-of-speech tagged fourgram	599.675	171.051	0.325	3.506	0.001	4.657
Bigram = Match degree of bigrams		Part-of-speech tagged bigram = Match degree of part-of-speech tagged bigrams				
Trigram = Match degree of trigrams		Part-of-speech tagged trigram = Match degree of part-of-speech tagged trigrams				
Part-of-speech tagged fourgram = Match degree of part-of-speech tagged fourgrams						

A combined analysis of the three genres’ regression models leads to the following two conclusions. First, among the three genres, the model fit was highest and most effective for the explanatory essay regression model, followed by the argumentative essay model and narrative essay model. The overall regression effect of the three genre models is good, and all of them can explain some translation scores to different degrees. Second, among the 2-4 phraseological units, the shorter the phrase length, the better its related variables performed in the three genre regression models. In all six variables, the bigram-related variables appeared the most frequently and were represented in all three genre regression models, with strong predictive power for the quality of translations in all genres. The trigram-related variables performed well in the explanatory and argumentative essay regression models. Among the fourgram-related variables, only the match degree of part-of-speech tagged fourgrams appeared in the argumentative essay regression model and was not reflected in the rest of the models.

By analysing all the data, it was found that there were two aspects that led to the above results. The first aspect is the specific characteristics of the three genres. The three genres are different, so their language expressions and linguistic characteristics are also different between the original language and the translated language. As demonstrated in the analysis of sentence complexity of the three genres in section 4.1, the language of explanatory essays is simple, while the argumentative essays are reasoning and logical. Both genres’ languages are concise. The language of narrative essays is vivid, rich in words and diverse in form. Therefore, the specific form and the quantity of 2-4 phraseological units extracted from the translations of the three genres and their degree of differentiation on the quality of the translations are different. The second aspect is the limitation of the N-gram match approach. Owing to the mechanical calculation of repeated phrases, the N-gram match approach fails to take into account the large number of synonymy and polysemy phenomena that exist in natural languages. In conclusion, due to the influence of the genre characteristics of the original text and the limitations of the N-gram match approach, the regression model of narrative essay is slightly weaker than that of explanatory essay and argumentative essay, and the regression model of explanatory essay is the most effective. In addition, the bigram-related variables have the strongest explanatory power for the translation scores, followed by the trigram-related variables, and the fourgram-related variables are the weakest.

## Conclusion

Under the view of phraseology, strictly following the definition and screening criteria of “phrase” in phraseology, this study quantitatively evaluated the translation quality from both semantic and formal aspects based on the N-gram match approach, which consisted of 6 relevant variables, including the match degree of 2-4 phraseological units and the match degree of 2-4 part-of-speech tagged phraseological units. The results of the study show that, firstly, the bigram-related variables and trigram-related variables are stronger than the fourgram-related variables in explaining the translation scores of the three genres; secondly, the overall fit of the translation quality assessment models of the three genres is good, among which the models of explanatory and argumentative essays are more effective and the model of narrative essays is somewhat weaker; thirdly, by combining the part-of-speech tagged phraseological units with the filtered phrases, the quality of translations can be evaluated from the perspective of both linguistic form and linguistic meaning, which can to some extent improve the prediction degree of phrases on the translation quality. At the phrase level, this study transformed the subjective assessment of translation quality into computer-recognizable and operable quantitative assessment criteria, and built translation quality assessment models for three genres.

The advent of artificial intelligence has caused a profound impact on the field of translation, giving rise to both challenges and opportunities. Translating is a frequently occurring component of subjective questions in a variety of English language assessments at all levels. This study offered novel perspectives and methodologies for translation quality assessment. It offers effective predictors for the quantitative assessment model of translation quality at the phrase level and promotes the optimization of the model. Conversely, the study also provided useful reference for the development of other automatic test scoring systems.

Despite the inherent limitations of the N-gram matching method, it could be addressed to a considerable extent by augmenting the number of reference translations in order to cover the potential N-grams. Nevertheless, further optimization in this domain remains a subject for future research. Potential avenues for enhancement include the utilization of large language models, the incorporation of synonym word forests into the scoring model through programming. The impact of linguistic phenomena, including polysemy and synonymy, on the assessment of translation quality could be circumvented so that the quantitative assessment model of translation quality can be optimized.

## Supporting information

S1 Data. Dataset (XLSX)
